# High sensitivity of HIV antibody screening tests may lead to longer time to diagnosis: a Case Report

**DOI:** 10.3389/fmed.2025.1562946

**Published:** 2025-05-15

**Authors:** Yuanfang Wang, Lan Luo, Jielun Deng, Xiaohan Li, Yi Xie, Dongdong Li

**Affiliations:** Division of Clinical Microbiology, Department of Laboratory Medicine, West China Hospital of Sichuan University, Chengdu, China

**Keywords:** HIV serological assay, P24 antigen, antibody HIV, diagnosis period, algorithm, fourth generation assay

## Abstract

**Background:**

The fourth-generation human immunodeficiency virus (HIV) serology assay, which simultaneously detects the HIV-1 p24 antigen and HIV-1 antibodies, is available either in a combined format or as dual tests that differentiate between the p24 antigen and antibodies. Divergent detection methodologies require distinct confirmatory testing algorithms, which significantly impact the time to HIV infection.

**Case presentation:**

In this report, we present three cases where the HIV-1 p24 antigen tested reactive, while the HIV-1 antibody remained non-reactive in a dual testing scenario—despite both the combined test and the colloidal gold immunochromatographic assay (GICA) for HIV-1 antibodies yielding reactive results. Upon further analysis of subsequent laboratory procedures, we observed that due to the application of various complementary tests, the assay with high antibody sensitivity such as the GICA paradoxically resulted in a prolonged time to diagnosis, extending the diagnostic window for patients from 5 days to 11 days.

**Conclusion:**

Our findings underscore the importance of prioritizing HIV-1 RNA testing in cases of discordant results between combined antigen/antibody testing, dual testing, and stand-alone antibody testing, particularly for patients who have not received pre-exposure or post-exposure prophylaxis.

## 1 Introduction

**Figure 1:**
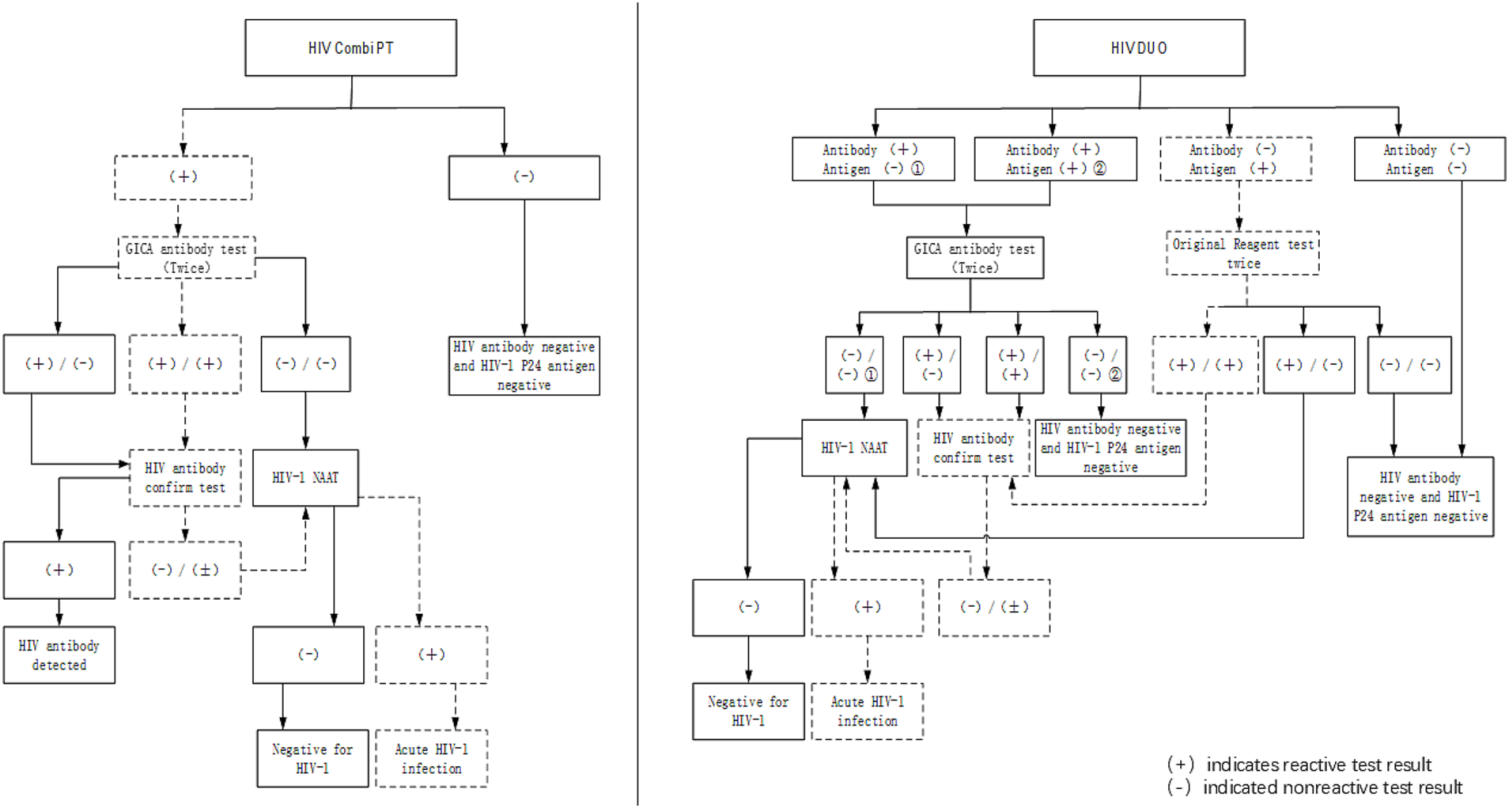
Two processing flows for HIV screening positive results. Elecsys^®^ HIV combi PT/ Elecsys^®^ HIV Duo and the GICA antibody test are used as screening procedures for HIV infection. HIV infection can be confirmed in patients with positive NAATs and positive confirmatory tests. The procedure of the Elecsys^®^ HIV combi PT- or Elecsys^®^ HIV Duo-positive supplementary test is shown in the figure. The dotted lines represent the processing flow for our three patients. Case 1 corresponds to the left one, and Cases 2 and 3 correspond to the right one. The HIV-1 antibody confirmatory test uses the Western blot technique to detect antibodies and is regarded as the most important supplementary test for HIV diagnosis in China because it can distinguish the type of HIV proteins targeted by antibodies in patients.

Human immunodeficiency virus (HIV) and acquired immunodeficiency syndrome (AIDS) are major public health problems worldwide. An HIV screening test is of great importance to identify all HIV-infected people and facilitate their linkage to care (1). The screening and detection of 90% of all HIV-infected people was declared a major goal by the UNAIDS (2). The fourth-generation antigen/antibody (Ag/Ab) immunoassay has become the most commonly used screening test due to its high sensitivity (3). Elecsys® HIV combi PT and Elecsys® HIV Duo are both fourth-generation reagents of electrochemiluminescence immunoassay (ECLIA). A critical distinction between the two assays lies in result interpretation: Elecsys® HIV combi PT yields a single composite result for the simultaneous detection of the p24 antigen and antibodies, whereas the Elecsys® HIV Duo provides discrete differentiation of antigen and antibody reactivity, thereby distinguishing whether a positive result is attributable to the presence of the HIV-1 p24 antigen or specific antibodies against HIV-1/2 (4). For samples reactive to HIV antibodies, the colloidal gold immunochromatographic assay (GICA) is routinely used as a rapid diagnostic test and employed as the retesting method. Concurrent positive results from both the GICA and the initial screening test generally indicate the need for a Western blot (WB) HIV-1 antibody confirmatory test, and a negative GICA result typically warrants supplemental HIV-1 RNA testing (nucleic acid amplification technologies, NAATs) to rule out acute HIV infection. The National Guideline for HIV/AIDS Detection (2020), issued by the Chinese Center for Disease Control and Prevention (5) (hereinafter referred to as the “Guideline”), stipulates distinct supplementary diagnostic algorithms (Figure 1) for these two types of test assays.

Here, we present three cases demonstrating reactivity in both Elecsys® HIV Duo and Elecsys® HIV combi PT independently. In one case, the diagnostic algorithm prompted GICA testing, which yielded reactive results; however, the time to definitive HIV diagnosis for this patient was nearly twice as long as that for the other two cases.

## 2 Case presentation

### 2.1 Case 1

A 43-year-old man was admitted to the emergency department with a fever lasting for 8 days, with the highest recorded temperature reaching 39°C. The inguinal lymph nodes had become enlarged 2 weeks earlier. An adequate, non-leaky qualified serum sample was sent for an HIV screening test. Since Elecsys® HIV combi PT (Roche Diagnostics, Germany, REF 05390095) was reactive (22.01 COI) and the GICA (Lizhu, China, REF20143401976) was positive, the patient was advised to undergo an HIV-1 antibody confirmatory test. Six days later, we obtained another sample from him for WB testing (MP Diagnostics, Singapore, REF 20163401575). Prior to testing, Elecsys® HIV Duo (Roche Diagnostics, REF 07229542190) was used as a conventional additional test according to our workflow.

**Figure 2:**
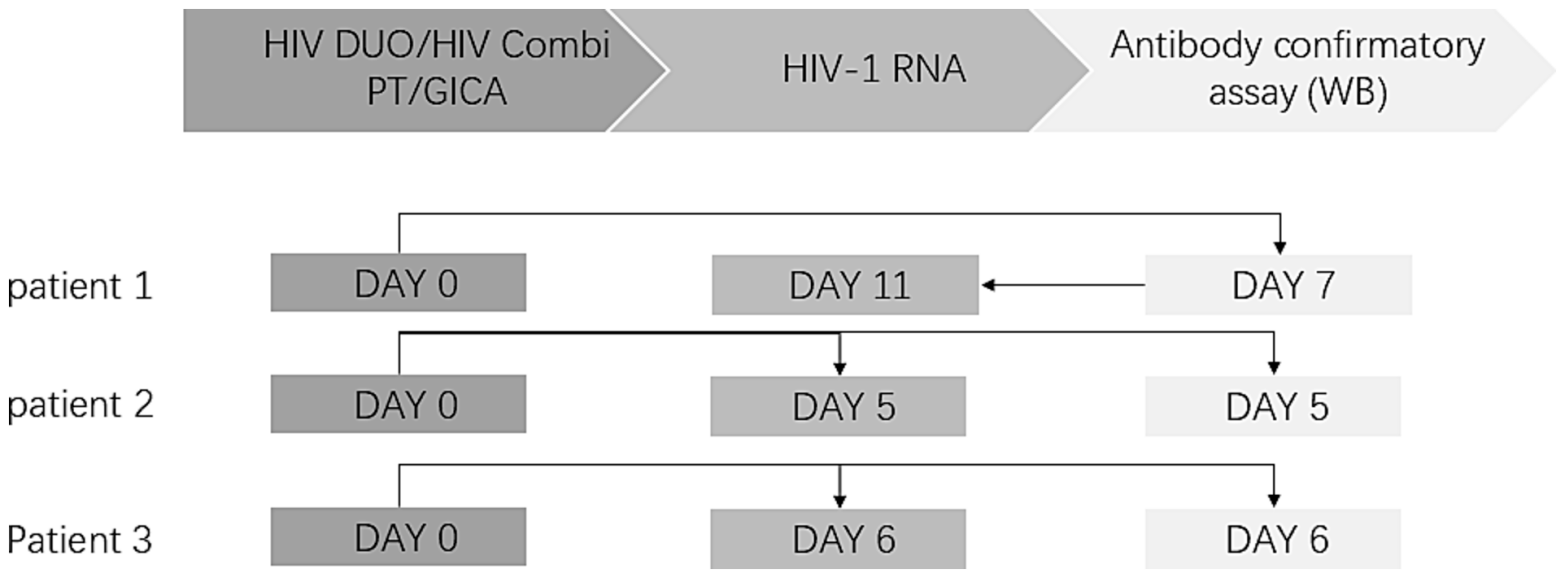
Diagnosis timeline of HIV patients. The laboratory diagnostic test procedure for HIV is divided into three sections: the HIV screening test, HIV RNA testing, and the HIV-1 antibody confirmatory test. The arrow indicates the sequence of the tests. In Cases 2 and 3, both HIV-1 RNA testing and Western blot (WB) antibody confirmation were submitted concurrently.

Notably, Elecsys® HIV Duo demonstrated discordant results compared to the GICA, and HIV antibodies were non-reactive (0.37 COI), whereas the p24 antigen exhibited reactivity (9.94 COI). The WB testing showed gp160 and p24 bands, which were classified as indeterminate. Given this discrepancy, confirmatory HIV-1 RNA testing was performed using the Roche cobas® system (REF 05212294190). Following a 4-day interval, subsequent testing revealed a high HIV-1 viral load of 2.86 × 10^6^ copies/mL, confirming the definitive diagnosis of HIV infection. In this patient, we found low CD4 + T-lymphocyte counts (BD, America, REF 34049) with 55 cells/μL. The diagnostic interval from the initial HIV screening test to the confirmed diagnosis was 11 days (Figure 2).

### 2.2 Case 2

A 46-year-old male was admitted to the otorhinolaryngology department with upper respiratory tract symptoms and lymphadenopathy. An HIV screening test was immediately conducted. Elecsys® HIV Duo demonstrated reactive p24 antigen (5.52 COI) with concurrent non-reactive HIV antibodies (0.35 COI). To prevent potential false-negative results (as demonstrated in Case 1), antibody retesting was conducted using the GICA, which yielded positive results. To verify this contradictory result, Elecsys® HIV combi PT was added as a third test, and the result was reactive (21.34 COI), prompting us to perform an HIV-1 antibody confirmatory test for this patient. However, in this case, we recommended the concurrent submission of two specimens: one for HIV-1 RNA testing and the other for the HIV-1 antibody confirmatory test. Four days later, the HIV-1 RNA test returned positive with a viral load of more than 1.00 × 10^7^ copies/mL, while the HIV-1 antibody confirmatory test was negative, showing no band. The patient was eventually diagnosed with HIV infection based on a positive HIV-1 RNA result. The diagnostic interval from the initial HIV screening test to the confirmed diagnosis was 5 days (Figure 2), CD4 + T-lymphocyte counts were also low, at a level of 78 cells/μL.

### 2.3 Case 3

A 46-year-old man with Guillain–Barre syndrome was admitted to the neurology department. Elecsys® HIV Duo showed reactive p24 antigen (8.95 COI) and non-reactive HIV antibodies (0.20COI). Similar to Case 2, retesting with the GICA and Elecsys® HIV combi PT showed positive results. To ensure a comprehensive diagnostic evaluation, we reiterated the recommendation for concurrent HIV-1 RNA testing and confirmatory HIV-1 antibody testing. As a result, the HIV-1 RNA testing was positive, with a viral load above 1.00 × 10^7^ copies/mL, while the WB testing was indeterminate, showing only the gp160 band.CD4 + T-lymphocyte counts were 712 cells/μL. This patient was also diagnosed with HIV infection. The diagnostic interval from the initial HIV screening test to the confirmed diagnosis was 6 days (Figure 2).

The studies involving human participants were reviewed and approved by the Ethics Committee of West China Hospital of Sichuan University. Informed consent was obtained from all patients.

## 3 Discussion

Serological tests for antigens and antibodies have been the most common method of HIV screening for a long time. Serological testing has evolved through four generations: the first generation used viral lysates for IgG antibody detection, the second generation employed recombinant antigens for IgG antibody detection, the third generation detected immunoglobulin M (IgM) and IgG antibodies, and the fourth generation detected IgM and IgG antibodies alongside the p24 antigen. As a fourth-generation reagent, Elecsys® HIV Duo shows good sensitivity and specificity. A multicenter evaluation of the reagent involving 13,328 blood donor samples showed a specificity of 99.87% (6). Elecsys® HIV Duo is considered to have slightly higher specificity (99.93% vs. 99.84%) and equivalent sensitivity compared to Elecsys® HIV combi PT (7). In a previous study involving 1,505 patients, the BioPlex 4th PLUS assay was assessed and found to have 100% sensitivity and 99.5% specificity (8).

The detection window of fourth-generation reagents is shorter, enabling serological detection within 5–7 days following a positive HIV-1 RNA test, as they can detect the HIV-1 p24 antigen. Approximately 1 week after the appearance of p24, immunoglobulin M (IgM) antibodies are detectable through third-generation immunoassays—several weeks earlier than first- or second-generation immunoassays that only detect immunoglobulin G (IgG) antibodies (9–12). Compared to Elecsys® HIV combi PT, Elecsys® HIV Duo can shorten the window period from 18 days to 14 days after HIV infection (13) because of its slightly different coating of antigens/antibodies, which reduces the proportion of cross-reactions. In addition, Elecsys® HIV Duo can avoid the secondary window period to a certain extent, while the window period for Elecsys® HIV combi PT is more difficult to minimize.

**Figure 3:**
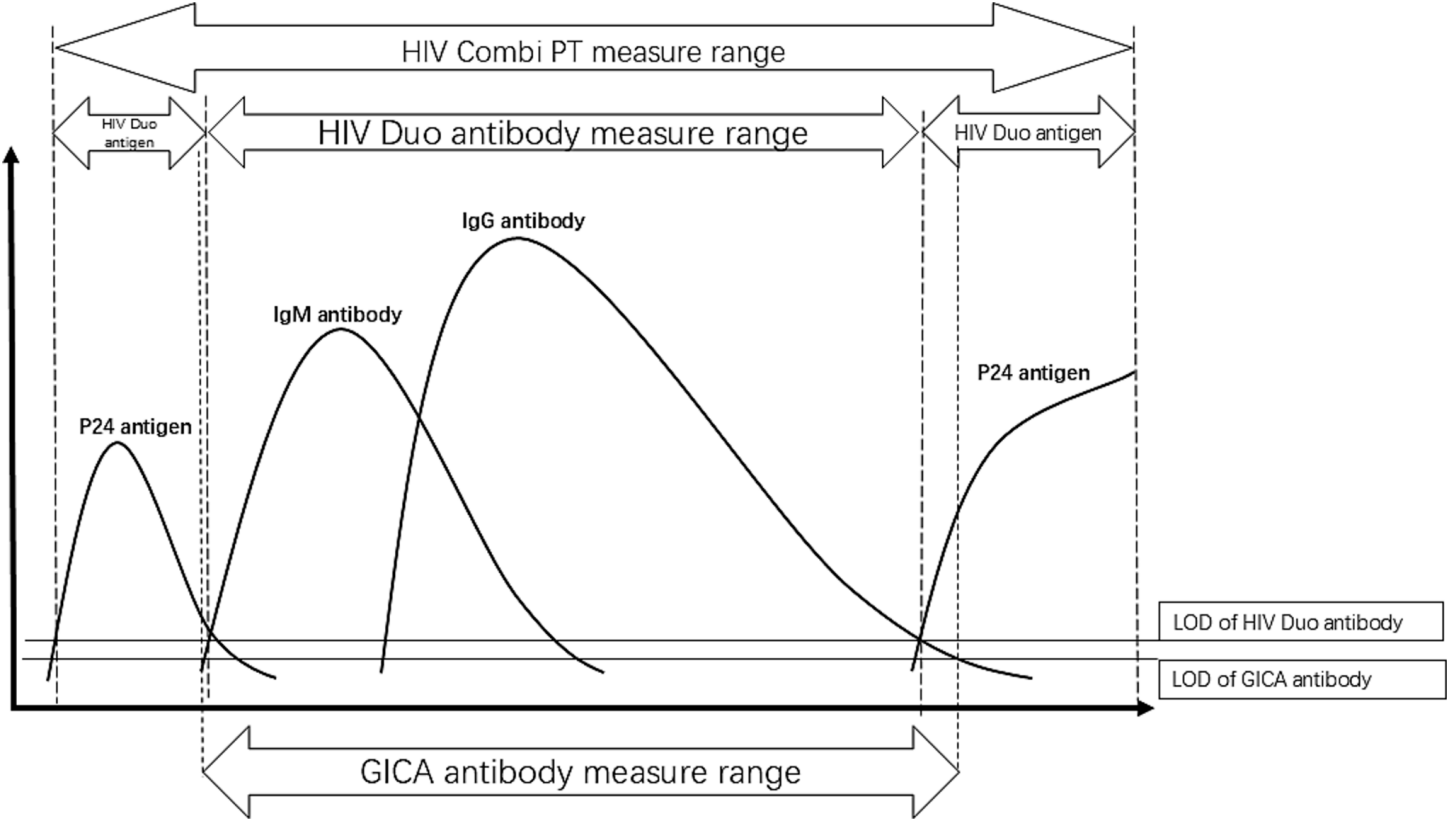
Pattern diagram of serological conversion of HIV infection. The dotted lines represent the detection range of different methods, while the solid lines indicate the detection limits of antigens or antibodies. The limit of detection (LOD) for the GICA antibody test is lower than that of the ROCHE HIV DUO antibody test. When the HIV Duo antibody test is negative, the GICA antibody test may be positive, while the HIV Duo antigen test will be positive, as observed in our cases. In the serological conversion process of HIV infection, this situation occurs during two phases—early infection and late infection. At these stages, different tests provide varying guidance. The HIV Duo antigen test takes precedence for patients whose antibodies are not detected, directing them to HIV RNA testing.

Given reports of HIV misdiagnoses associated with fourth-generation reagents, to avoid false-positive antibody results from Elecsys® HIV Duo and Elecsys® HIV combi PT (3, 14), third-generation reagents such as the GICA with lower detection limits are used as supplementary methods in screening tests. Although only IgG antibodies are detected, due to the high affinity of gp41 and gp120, the GICA reagent has a lower limit for antibody detection than fourth-generation reagents. It usually yields a positive result at the same time as, or earlier than, WB testing (15, 16) (Figure 3). Samples that test positive by ECLIA, the GICA, and WB testing will be used as direct evidence for an antibody-based HIV diagnosis. In contrast, only ECLIA-positive samples will prompt HIV-1 RNA testing (Figure 1). Due to its high specificity and ability to evaluate the stage of infection based on the separation of HIV-1 viral proteins by molecular weight, the WB technique has long been used as a confirmatory assay. However, the HIV-1 Western blot test still relies on first-generation principles, using whole viral lysate as the source of antigens and an enzyme-conjugated anti-IgG to bind to individual HIV proteins. The long detection window period makes it easy to prolong the diagnosis time. HIV-1 RNA is considered the earliest detectable marker of HIV infection and can be detected 7–10 days after infection, even when antigen and antibody tests are still negative (11, 17). It can be detected using PCR or NAATs with high sensitivity. HIV-1 RNA positivity is generally considered direct evidence of HIV infection. However, the risk of false-negative results remains due to viral replication inhibition by antiretroviral therapy and pre-exposure prophylaxis/post-exposure prophylaxis (18–20).

Across all three cases in our series, we observed that, as an antibody supplemental test, the GICA results were inconsistent with the antibody testing results from Elecsys® HIV Duo. Among patients with reactive GICA results, it took much longer for HIV infection to be diagnosed in patients who continued with confirmatory WB testing only (Case 1). We believe that the high sensitivity of the GICA led to reactive results, which subsequently directed patients to WB testing, a method with longer window periods. It is worth noting that in Case 2, the likelihood of missing the diagnosis would have been high if we had not performed additional HIV-1 RNA testing and only performed HIV antibody confirmatory testing based on the supplemental testing procedures of Elecsys® HIV combi PT. Our study demonstrated that incorporating higher-sensitivity antibody detection assays into the initial screening protocol failed to significantly improve diagnostic timeliness. Crucially, HIV-1 RNA testing remained indispensable regardless of the reactivity status in subsequent antibody screening. Furthermore, to preclude the influence of pre-exposure or post-exposure prophylaxis on HIV-1 RNA testing, WB testing is essential and should be conducted concurrently.

In conclusion, our patients presented with unusual cases of discrepant HIV antibody screening results, which led to different recommendations and affected the time to diagnosis and the assessment of infection status. Higher sensitivity antibody screening results lead to HIV-1 antibody confirmatory testing, which, in turn, leads to longer diagnostic times. Therefore, even when the screening procedure confirms HIV antibody reactivity, HIV-1 RNA testing needs to be performed concurrently.

## Data Availability

The data analyzed in this study is subject to the following licenses/restrictions: the dataset contains patient information. Requests to access these datasets should be directed to Yuanfang Wang, 180402617@qq.com.
